# Antithrombotic Treatment Patterns of Patients with Symptomatic Peripheral Arterial Occlusive Disease in Germany: Evidence from Health Insurance Claims Data

**DOI:** 10.3390/jcm11185455

**Published:** 2022-09-16

**Authors:** Frederik Peters, Jenny Kuchenbecker, Laura Acar, Ursula Marschall, Helmut L’Hoest, Fabien Lareyre, Konstantinos Spanos, Christian-Alexander Behrendt

**Affiliations:** 1 Research Group GermanVasc, University Medical Centre Hamburg-Eppendorf, 20246 Hamburg, Germany; 2 BARMER, 42266 Wuppertal, Germany; 3 Department of Vascular Surgery, Hospital of Antibes Juan-les-Pins, 06600 Antibes, France; 4 Vascular Surgery Department, University Hospital of Larissa, Faculty of Medicine, School of Health Sciences, University of Thessaly, 41222 Larissa, Greece; 5 Brandenburg Medical School Theodor Fontane, 16816 Neuruppin, Germany; 6 Department of Vascular and Endovascular Surgery, Asklepios Clinic Wandsbek, Asklepios Medical School, 22043 Hamburg, Germany

**Keywords:** peripheral arterial occlusive disease, chronic limb-threatening ischaemia, intermittent claudication, antithrombic drugs, health insurance claims data

## Abstract

Objectives: Patients with peripheral arterial occlusive disease (PAOD) are at risk of worsening limb symptoms, major adverse cardiovascular events and exhibit an impaired life expectancy. There is a lack of evidence on the extent of pharmacological secondary prevention in PAOD patients. This study assesses treatment patterns of antithrombotic agents in symptomatic PAOD patients. Methods: This is a retrospective cohort study using data from the second largest insurance fund in Germany, BARMER. We included symptomatic PAOD patients undergoing in-hospital treatment with an index admission between 1 January 2010 and 31 December 2017. Outcomes were proportions of single antiplatelets (SAPT), dual antiplatelets (DAPT), vitamin-K antagonists (VKA), or direct oral anticoagulants (DOAC) in the 12 months prior and 6 months after the index hospitalization. Non-parametric cumulative incidence for competing risks was estimated to account for censoring and death after discharge from hospital stay. Patient flows were visualised by alluvial diagrams. All analyses were stratified by intermittent claudication (IC) and chronic limb-threatening ischaemia (CLTI). The protocol was registered to ClinicalTrials.gov (NCT03909022). Results: A total of 80,426 unique patient encounters were identified. Mean age was 72.7 (46.3% female). Amongst all patients, 25.6% were on SAPT, 4.1% on DAPT, 9.1% on VKA, 3.9% on DOAC, 3.9% on both antiplatelets and oral anticoagulation, and 53.3% without any antithrombotic therapy during the 12 months before index stay. The estimated cumulative incidence was 37.9% SAPT, 14.8% DAPT, 7.5% VKA, 4.3% DOAC, 7.4% both, and 28.1% without any antithrombotic therapy during the 6 months after index stay. The considerable increases in antiplatelet therapy were mainly driven by the group of patients without antithrombotics before index stay. As compared with IC, patients who suffered from CLTI received less often antiplatelets but more often anticoagulants both before and after index stay. Conclusions: Utilisation rates of antithrombotic therapy increased considerably after in-hospital treatment for PAOD. Yet, remarkably high rates of symptomatic patients without any blood-thinning therapy constitute a major concern with respect to adequate secondary prevention of PAOD patients.

## 1. Introduction

Peripheral arterial occlusive disease (PAOD) is predominantly caused by atherosclerotic obstruction of the peripheral arteries of the lower extremities, which results in a lack of blood flow to the lower limbs and subsequent ischaemia affecting approximately 237 million patients worldwide [1]. In its symptomatic form, PAOD results in intermittent pain or discomfort of the limb, termed as intermittent claudication (IC). If the obstruction becomes severe, patients may experience chronic limb-threatening ischaemia (CLTI), as indicated by ulceration, gangrene, or ischaemic rest pain [2,3,4].

Patients with symptomatic PAOD are at high risk of worsening limb symptoms or of major adverse cardiovascular events (MACE) and face an impaired life expectancy [5]. Guideline recommendations refer to single antiplatelet therapy (SAPT) with clopidogrel or aspirin as a cornerstone of secondary prevention in symptomatic PAOD patients [6]. More potent antithrombotic therapy involving multiple antiplatelet agents, anticoagulation, or even combinations of these agents were recommended for selected patients with more severe disease progression, repeated revascularisation, or concomitant indication for anticoagulation (e.g., atrial fibrillation or venous thromboembolism).

While there is a heated discussion about the safety and effectiveness of more intensive treatment options, little is known about real-world utilisation of different treatment regimens [7,8,9]. Existing studies on secondary prevention in Western countries documented a striking undertreatment with often less than half of PAOD patients receiving any antiplatelet agent [10,11,12,13,14,15]. Studies on invasively treated subgroups with more advanced disease as well as studies in East Asia reported higher coverage concerning antiplatelet therapy [16,17,18]. Yet, there is a lack of high-quality evidence on the distribution of utilisation of single and dual antiplatelet therapy and combination with anticoagulation, especially quantifying the group of patients without antithrombotic therapy.

Further contributing to the complexity of this research topic, there is a close connection between PAOD and concomitant diabetes. Thereby, the latter may be associated with more complex calcified lesions below the knee. While there is a widespread of endovascular therapies, restenosis and thrombosis remain their Achilles heel emphasising the urgent need for an optimal antithrombotic strategy after revascularisation.

Using nationwide and unselected German health claims data, the goal of this study was to assess the proportion of patients with SAPT, dual antiplatelet therapy (DAPT), vitamin-K antagonists (VKA), direct oral anticoagulants (DOAC), and dual pathway inhibition (DPI) twelve months prior and six months after the index hospitalisation for symptomatic PAOD.

## 2. Materials and Methods

### 2.1. BARMER Cohort

The health insurance claims data of Germany’s second largest insurance provider, BARMER, includes the outpatient and inpatient medical care provided to approximately 9.4 million German citizens (13.2% of Germany’s population). The BARMER cohort is similar to Western European countries, has been widely used for research projects before and contains validated information about diagnoses, in-hospital treatment and pharmacological therapy [19,20,21,22,23,24].

We used the German adaptation of the International Classification of Diseases (ICD-10-GM) to identify diagnoses and Operations and Procedures Codes (OPS) coding to identify procedures. The German OPS code is adapted to the International Classification of Procedures in Medicine (ICPM). For identifying medical prescriptions, the German version of the international Anatomical Therapeutic Chemical (ATC)-Classification was utilized. This study is part of a larger project on assessing best medical treatment of PAOD patients. Further details could be found in the published study protocol (clinicaltrials.gov NCT03909022), where also recent international guideline recommendations concerning antithrombotic therapy were summarized [25]. The precise analyses in this paper were tailored to the specific research question on details of antithrombotic treatment.

### 2.2. Study Population

This study is a retrospective analysis of nationwide inpatient data. Patients ≥18 years of age with an index stay (including hospital ambulance records, excluding patients in partial hospital care) for symptomatic PAOD between 1 January 2010 and 31 December 2017 were eligible for inclusion in the study. As a sensitivity analysis, patients with the following indications for anticoagulation were also excluded: atrial fibrillation (AF, within 1 year prior to index date), venous thromboembolism (VTE, within 60 days prior to the index date), acute coronary syndrome (ACS, within 1 year prior to the index date) and hip or knee replacement (HKR, within 60 days prior to the index date).

Index stay denotes the first symptomatic PAOD-related in-hospital admission within 5 years (using data dating back to 1 January 2005). To assess post-discharge prescription status adequately, we excluded patients that were referred to a hospice or died during hospital stay or up to 6 months after discharge (using data up to 30 June 2018). We included only patients that were continuously insured at BARMER during the 5 years before index stay.

Symptomatic PAOD patients were identified using ICD-10-GM codes of primary or secondary diagnosis during in-hospital stay (for coding see web supplement Table S1).

If PAOD was coded as secondary diagnosis only, inclusion additionally required a primary diagnosis of: 1. type 1 or type 2 diabetes with peripheral vascular complications, or with multiple complications including diabetic foot syndrome, 2. other peripheral vascular diseases, 3. arterial embolism and thrombosis, 4. cellulitis of finger and toe including acute lymphangitis, or 5. chronic ulcer of skin and gangrene, not elsewhere classified (for coding see web supplement Table S1) [26]. The ICD codes were grouped according to Fontaine stages II (IC), III (ischemic rest pain) and IV (for ischemic ulcers or gangrene) representing different degrees of severity of PAOD [27].

### 2.3. Study Variables

The primary objective of this study is to assess the proportion of patients with antithrombotic therapy prior and after to the index hospitalisation. Classification of treatment patterns was performed according to the ESC 2017 guidelines [6].

Treatment groups were defined as follows. Therapy with SAPT: only prescription for aspirin (ATC code: B01AC06, B01AC56, B01AC86) or only prescription for clopidogrel (ATC code: B01AC04) and no prescription for any anticoagulation therapy (ATC code: B01AA, B01AE, B01AF). Therapy with DAPT: at least one prescription for aspirin and at least one prescription for clopidogrel or clopidogrel/aspirin dual prescription (ATC code: B01AC34) and no prescription for anticoagulation therapy. Therapy with VKA: at least one prescription for VKA (ATC code: B01AA) and no prescription for DOAC. Therapy with DOAC: at least one prescription for DOAC (ATC code: B01AE, B01AF). Therapy with DPI: Therapy with both, antiplatelet and anticoagulation therapy. In case of receiving none of the treatments listed above, patients were grouped as no therapy. The definition of these groups mutually exclusive so that every patient can only be assigned to one of the groups. We excluded patients not receiving any of the agents defined in the groups above, but other antithrombotic treatment not recommended by guidelines for secondary prevention.

Baseline characteristics were sex, age, obtained in-hospital treatment at index date (best medical treatment, peripheral vascular intervention, open surgical revascularisation, minor or major amputation only). If multiple treatments are coded at one hospital stay, we grouped the categories hierarchically in open surgical revascularisation, peripheral vascular intervention, amputation and best-medical-treatment. Clinically relevant variables were obtained from current and previous in-hospital diagnoses (lookback 5 years) and measured according to the 30-item list of Elixhauser and colleagues translated to ICD-10-GM codes and summarized to the van Walraven score, ranging from −19 to +89 (grouped in 0–4, 5–9, 10+) [28]. We presented 9 of the 30 Elixhauser conditions in the baseline table: congestive heart failure, cardiac arrhythmias, valvular disease, hypertension, diabetes (complicated), renal failure, liver disease, solid tumour or metastatic cancer complemented by five non-Elixhauser items dyslipidaemia, history of coronary artery disease, prior myocardial infarction, prior stroke/transient ischaemic attack (TIA), and history of bleeding (for coding see electronic supplement Table S1).

### 2.4. Statistical Analysis

Counts and proportions of patients in one of the treatment groups were assessed 12 months prior to the index hospitalisation and 6 months after discharge. Patient characteristics are presented in percentage for categorical variables and mean (+standard deviation) for continuous variables based on visual inspection of the respective distributions. To account for censoring and death during the 6 months after hospital discharge, we computed cumulative prescription incidence with 95% confidence intervals assuming independent competing risks based on the Aalen-Johansen estimator and the corresponding asymptotic variance suggested by Aalen [29].

For this analysis, all patients discharged from hospital were followed until either their first filling of a drug prescription, loss to follow-up due to death or change to another health insurance or end of follow-up after 6 months. To visualise the underlying flows responsible for the change in the distribution of treatment patterns before and after index stay, we employed alluvial diagrams. For the sake of clarity and traceability of patient flows, SAPT and DAPT were grouped to antiplatelet therapy (APT) and VKA and DOAC were grouped to oral anticoagulation therapy (OAC). Here, only patients alive and fully insured up until 6 months after discharge from index stay were included.

All analyses were computed for the full sample and stratified by chronic limb-threatening ischaemia (Fontaine stages III + IV) and intermittent claudication (Fontaine stage II). We excluded missing or implausible information before analysing the data (<0.5% of observations). All analyses were performed with software SAS version 9.04 (SAS Institute, Cary, NC, USA) and R version 3.6.0. (packages: cmprsk (version 2.2–11) and alluvial (version 0.1–2)). The reporting of this study adhered the reporting of studies conducted using observational routinely collected health data (RECORD) statement [30] and good practice of secondary data analysis [31].

## 3. Results

**Figure 1:**
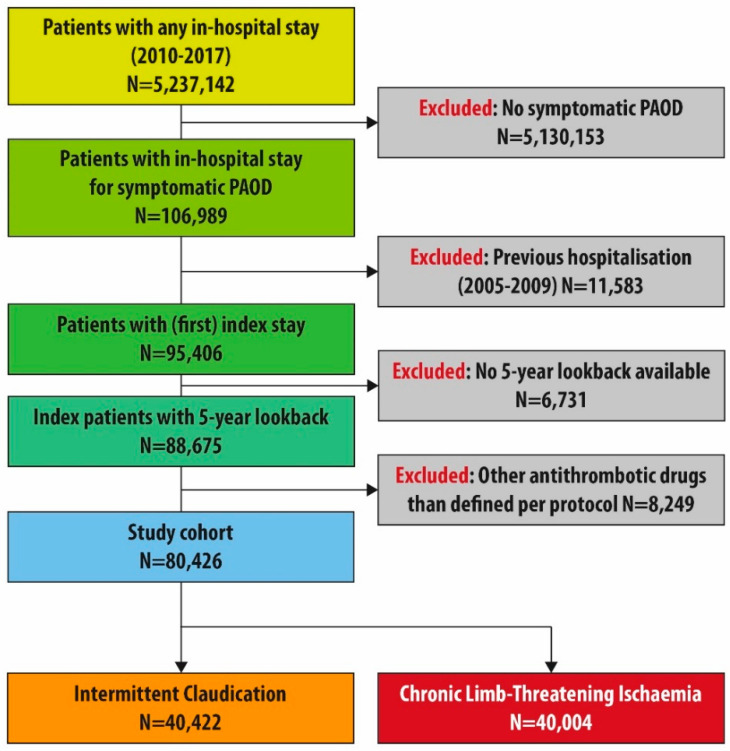
Flow chart of the study cohort, PAOD: Peripheral arterial occlusive disease.

We included 80,426 patients with index stay for symptomatic PAOD between 2010 and 2017 in our study (see flow chart Figure 1) with a mean age of 72.7 ± 11.3 years and a share of 46.3% females. About half of all patients were suffering from CLTI at index stay (N = 40,004). Within 6 months after discharge from index stay in total 8854 patients died (11.0%), which was 7777 in CLTI patients (19.4%) and 1077 in IC patients (2.7%).

### 3.1. Patient Characteristics

At their index stay, about half of all PAOD patients were treated with a peripheral vascular intervention (49.0%), while amputation only (without revascularisation) was performed in 4.8% of the cases (see Table 1). Most prevalent chronic conditions were hypertension (82.4%), dyslipidaemia (49.0%) and history of coronary artery disease (37.1%), while liver disease (5.0%) and cancer (8.0%) were less frequent.

Patients with CLTI underwent peripheral vascular intervention less often than IC patients (37.3% vs. 60.5%) and had a less favourable comorbidity profile (van Walraven score > 9 points: 55.6% vs. 25.4%) except for dyslipidaemia (CLTI: 45.4% vs. IC: 52.5%). CLTI patients were particularly susceptible for amputation at index stay (9.4%), congestive heart failure (40.7%), cardiac arrhythmias (40.4%), complicated diabetes (41.4%), renal failure (43.1%) and a history of bleeding (33.9%) as compared to IC patients.

### 3.2. Baseline Characteristics of the Subgroup Excluding Patients with Concomitant Indication for Anticoagulation (Sensitivity Analysis)

Excluding patients suffering from atrial fibrillation within 1 year before the index date, venous thromboembolism within 60 days before the index date, acute coronary syndrome within 1 year before the index date and hip or knee replacement within 60 days before the index date, reduced the sample to 28,234 CLTI patients (−29.4%) and 35,909 IC patients (−11.2%). In this reduced sample, proportionally fewer CLTI patients were suffering from cardiological conditions such as congestive heart failure, cardiac arrhythmias, valvular disease and a history of coronary artery disease (see web supplement Table S2).

### 3.3. Treatment Patterns in the Entire Cohort

12 months before the index stay, 25.6% of all patients received SAPT, 4.1% DAPT, 9.1% VKA, 3.9% DOAC and 3.9% DPI, while 53.3% did not receive any antithrombotic therapy during the 12 months before index stay (see Table 2). During the 6 months after index stay, the proportion of patients receiving SAPT, DATP and DPI increased markedly to 37.9%, 14.8% and 7.5%, while therapy with VKA slightly decreased to 7.5% and therapy with DOAC increased to 4.3%. The share of patients without any antithrombotic therapy almost halved to a level of 28.1%.

Before index stay, antiplatelet treatment patterns of CLTI and IC patients were broadly similar while slightly more CLTI patients received anticoagulation: (VKA 12.8% vs. 5.5%, DOAC 5.3% vs. 2.6% and DPI 5.0% vs. 2.7%). For this reason, CLTI patients were less often among the group without any antithrombotic treatment as compared to IC patients (57.9% vs. 48.7%).

After index stay, both CLTI and IC patients exhibited considerable increase in antiplatelet utilisation, even more expressed in IC patients, while the proportion of patients with anticoagulation remained fairly stable. In both subgroups, the remaining share of patients without any antithrombotic therapy was about 28%.

In the reduced sample, where patients with an indication for anticoagulation were excluded (see web supplement Table S3), prescription rates with such agents were markedly lower with less than 4% of patients receiving VKA and less than 2% of patients receiving DOAC. By contrast, the share of patients with antiplatelets and patients without any antithrombotic therapy was slightly higher than in the full sample.

**Figure 2:**
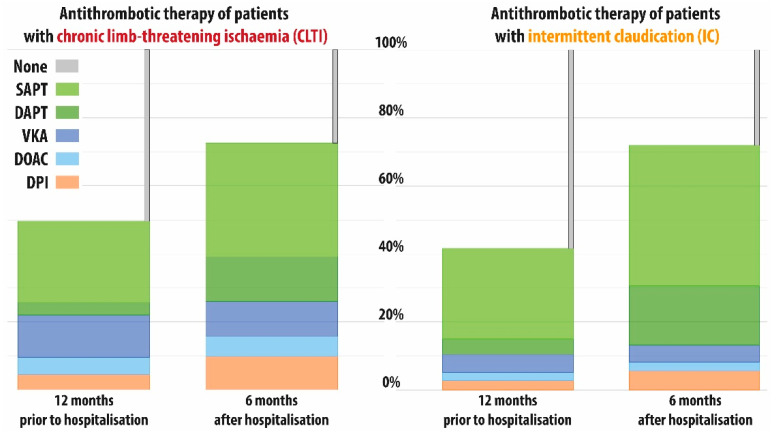
Proportions of patients with antithrombotic therapy 1 year before and 6 months after index stay for symptomatic peripheral arterial occlusive disease (PAOD); SAPT: Single antiplatelet therapy, DAPT: Dual antiplatelet therapy, VKA: Vitamin K antagonists, DOAC: Direct oral anticoagulation, DPI: Dual pathway inhibition, CLTI: Chronic limb-threatening ischaemia, IC: Intermittent claudication.

**Figure 3:**
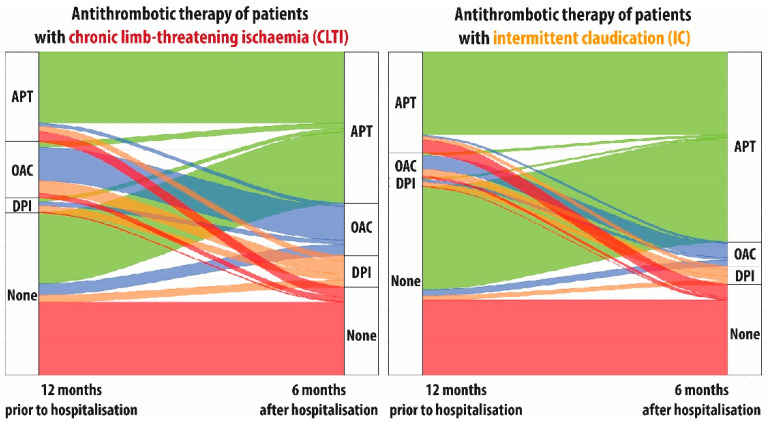
Alluvial diagram of proportions of patients with antithrombotic therapy 12 months before and 6 months after index stay for symptomatic peripheral arterial occlusive disease (PAOD). The different colours refer to the subgroups described at the right axis; APT: Antiplatelet therapy, OAC: Oral anticoagulation, DPI: Dual pathway inhibition, CLTI: Chronic limb-threatening ischaemia, IC: Intermittent claudication; only patients alive and fully insured up until 6 months after discharge from index stay were included (N = 71,794).

Overall, the change in the distribution of antithrombotic treatment patterns at index stay was mainly driven by considerable increases in therapy with SAPT and DAPT while even after index stay merely about 70% of all patients received any antithrombotic treatment (see Figure 2). Analysing these changes in more detail reveals that about half of patients without antithrombotic therapy before index stay received antiplatelet treatment afterwards, while only a small fraction of these patients were put on OAC or DPI therapy (see Figure 3). Interestingly, a sizeable fraction of patients receiving antiplatelets before index stay, did not receive any treatment during the 6 months after discharge. Utilisation of DPI after index stay was mainly driven by OAC users before the index stay.

## 4. Discussion

This is the first large-scale population-based analysis providing an overview of different antithrombotic strategies as defined in current practice guidelines in patients suffering from symptomatic PAOD in a real-world setting. We found that after index stay for symptomatic PAOD, mild antithrombotic therapy with SAPT and more intense antithrombotic therapy with a combination of DAPT and anticoagulation was administered in slightly more than a third of patients, respectively. Slightly less than a third received no antithrombotic therapy at all after being discharged. The considerable increase in antiplatelet therapy after index stay when compared with before index stay was mainly driven by patients not receiving any antithrombotics before. A more aggressive therapy was more common amongst patients with CLTI than IC and, interestingly, the share of patients without any antithrombotic therapy did not differ between these groups. Thereby, a concomitant indication for anticoagulation unrelated to PAOD (e.g., atrial fibrillation) explained almost completely the utilisation of such agents in the current cohort.

As compared with previous studies on drug utilisation (see web supplement Table S4), mostly providing lists of various (overlapping) drug agents, the current study provided more fine-grained and meaningful treatment schemes, where groups of patients were defined mutually exclusive.

The question arises why antithrombotic therapies are consistently underused in patients with symptomatic PAOD compared to patients with cardiac or cerebral arterial diseases. The reasons for that are likely multifactorial involving insufficient awareness amongst both general practitioners and patients as well as a general lack of high-level comparative effectiveness evidence on antithrombotic therapies. Furthermore, the high proportion of multiple comorbidities and frailty syndrome in this target population may drive the considerably low prescription rates since polypharmacy and certain comorbidities may be considered as contraindication by prescribing physicians in outpatient facilities [32,33,34,35].

Societal practice guidelines recommended antiplatelets as first-line treatment in patients with symptomatic PAOD to help to prevent adverse limb-related or cardiovascular events [6,36]. Against this background, the remarkably high rates of patients without any antithrombotic therapy before and after index stay for symptomatic PAOD constitute a major concern with respect to secondary prevention. Unlike the other drugs studied in the current study, low-dose aspirin was sold only in registered pharmacies but also obtainable without a prescription (over the counter). To date, however, it remains uncertain how often patients choose to buy aspirin instead of filling their prescription. Hence, the prevalence of antiplatelet use in the current study might be higher, possibly affecting the estimation of SAPT, DAPT and DPI. For this reason, the estimates should be seen more as lower bound of the true proportion.

A prospective cohort study (NCT03098290) and secondary analyses of the GermanVasc study data is currently surveying patients on that issue to understand how many patients are missing in administrative data on prescriptions. Yet, over-the-counter sales are less of an issue for PAOD patients since more recent guidelines favoured clopidogrel over aspirin, which is only available by prescription. In addition, those patients most likely received more than one medication making it less presumable that only aspirin among other medications would be obtained over the counter.

Based on screening at a personal visit, Galas et al. reported use of any antiplatelets in only half of 357 residents of nursing homes in Germany suffering from PAOD [11]. Additionally, a recently published study on the same patient cohort as ours reported large groups of patients without adequate best pharmacological treatment also for statins and antihypertensives available on prescription only [37]. A validation study in the UK reported that over-the-counter sales had only negligible impact on analyses of low-dose aspirin use in routine data [38]. In sum, we believe that a sizable group of PAOD patients does indeed not receive any antithrombotic treatment similarly than in other Western countries [13,14,15,39].

The share of patients without antithrombotic treatment was surprisingly similar among CLTI and IC patients at about 28%, respectively. Particularly CLTI patients are at high risk of adverse cardiovascular or limb events, and death [7]. The Critical Leg Ischaemia Prevention Study demonstrated that low-dose aspirin reduced the incidence of vascular events by 26% [40]. Since a large fraction of CLTI patients also suffers from obesity, the prescription gap for antiplatelet therapy might be related to a presumed aspirin resistance in this subgroup [41].

About 34% of patients received more intense antithrombotic therapy, where in 19.2% oral anticoagulation was involved, which went down to about 9% after excluding patients with venous thromboembolism, acute coronary syndrome and hip or knee replacement. Since the index stay for symptomatic PAOD hardly influenced this proportion, we believe that most patients received anticoagulation for other reasons, in line with guideline recommendations discouraging anticoagulation therapy in general PAOD patients for the sole purpose of secondary prevention [6,7]. Our estimates are in line with prior studies, where the proportion of anticoagulation ranges between 4% and 15% (see web supplement Table S4) [11,42]. We cannot rule out whether the slightly higher share in our study might reflect a general trend towards a more intensive treatment or higher share of patients with an indication for such treatment.

Although hardly emphasised by valid practice guidelines beyond very few special indications, dual pathway inhibition was administered in about 7% of patients, which was two per cent higher in CLTI patients. Interestingly, recent industry-sponsored trials suggested additional potential benefits of a combination of low-dose aspirin therapy and low-dose anticoagulation for secondary prevention [43,44]. Yet, the potential net benefit including adverse cardiovascular as well as major bleeding events, both potentially fatal, need to be assessed thoroughly and on a patient-individual level before taking such options into account. While it is commonly known that randomised trials may be affected by numerous biases, real-world data from clinical and administrative registries may complement the knowledge base especially for long-term and rare outcomes, which has proven useful in comparable cases [45]. Most recently, two machine learning predictive risk models were developed and externally validated to estimate the probability of limb-related and major bleeding events in patients with symptomatic PAOD, while the long-term event rates appeared likewise sobering in this target population (https://score.germanvasc.de, accessed on 15 September 2022) [46,47,48].

Using longitudinal information, we were able to measure flows of drug utilisation before and after index stay in more detail. Sigvant et al. reported that the in-hospital treatment in Swedish PAOD patients affected particularly antiplatelet use, more pronounced in IC than in CLTI patients [49]. Our study confirmed these findings for Germany, adding that the increase was greatest for SAPT followed by DAPT and DPI. It is concerning that a non-negligible fraction of patients on antiplatelets before index stay did not receive any prescription for these agents afterwards.

Our study has several strengths but also limitations. Although claims data contain detailed and objective information on medical therapy, they do not contain more specific aspects on indication such as laboratory values for assessing whether treatments were adequate. Further, we did not cover the aspect of adherence but rather just assessed whether a specific agent was prescribed at least once within a specific time frame. For the same reason, we cannot verify whether multiple agents, e.g., DAPT, were taken simultaneously or sequentially. Finally, our study is covering patients filling their prescription in a pharmacy after being discharged from the hospital. We cannot rule out that a certain fraction of patients refrained from taking the drug afterwards. Since our data included only patients up until 2017, our study could not assess how the increased awareness for the treatment of PAOD and new treatment options may influenced treatment patterns.

Future studies may take these aspects into account.

In 2023, for the first time, a practice guideline will be published by the European Society for Vascular Surgery (ESVS) that is exclusively dedicated to the antithrombotic therapy of vascular disease.

## 5. Conclusions

Although index stay for symptomatic PAOD markedly improved pharmaceutical secondary prevention in PAOD patients, the remarkably high rates of patients without any such therapy constitute a major concern with respect to secondary prevention. Yet, the fraction of over-the-counter use of low-dose aspirin needs to be addressed in further research in more detail.

## Figures and Tables

**Table 1:** Baseline characteristics of the total study cohort and CLTI and IC patients. SD: Standard Deviation; TIA: Transient ischaemic attack; CLTI: Chronic limb-threatening ischaemia, IC: Intermittent Claudication.

	Total (N = 80,426)	CLTI (N = 40,004)	IC (N = 40,422)
Age, years, mean (SD)	72.74 (11.26)	75.98 (11.37)	69.53 (10.19)
Female sex, n (%)	37,258 (46.3)	19,478 (48.7)	17,780 (44.0)
van Walraven Score > 9, n (%)	32,539 (40.5)	22,260 (55.6)	10,279 (25.4)
No invasive treatment at index, n (%)	17,488 (21.7)	11,635 (29.1)	5853 (14.5)
Percutaneous endovascular intervention, n (%)	39,382 (49.0)	14,929 (37.3)	24,453 (60.5)
Open surgical repair, n (%)	19,658 (24.4)	9668 (24.2)	9990 (24.7)
Primary amputation only, n (%)	3898 (4.8)	3772 (9.4)	126 (0.3)
Congestive heart failure, n (%)	23,516 (29.2)	16,272 (40.7)	7244 (17.9)
Cardiac arrhythmias, n (%)	23,987 (29.8)	16,174 (40.4)	7813 (19.3)
Valvular disease, n (%)	10,117 (12.6)	6783 (17.0)	3334 (8.2)
Hypertension, n (%)	66,233 (82.4)	33,976 (84.9)	32,257 (79.8)
Diabetes (complicated), n (%)	23,154 (28.8)	16,547 (41.4)	6607 (16.3)
Renal failure, n (%)	25,940 (32.3)	17,239 (43.1)	8701 (21.5)
Liver disease, n (%)	4057 (5.0)	2554 (6.4)	1503 (3.7)
Solid tumour or metastatic cancer, n (%)	6421 (8.0)	3594 (9.0)	2827 (7.0)
Dyslipidaemia, n (%)	39,371 (49.0)	18,158 (45.4)	21,213 (52.5)
History of coronary artery disease, n (%)	29,873 (37.1)	16,255 (40.6)	13,618 (33.7)
Prior myocardial infarction, n (%)	12,084 (15.0)	6737 (16.8)	5347 (13.2)
Prior stroke or TIA, n (%)	8088 (10.1)	5336 (13.3)	2752 (6.8)
History of bleeding, n (%)	20,182 (25.1)	13,543 (33.9)	6639 (16.4)

**Table 2:** Observed and estimated treatment patterns of the total study cohort, CLTI and IC patients; SAPT: Single antiplatelet therapy, DAPT: Dual antiplatelet therapy, VKA: Vitamin-K-antagonists, DOAC: Direct oral anticoagulation, DPI: Dual pathway inhibition, IC: Intermittent claudication, CLTI: Chronic limb-threatening ischaemia, CI: Confidence interval, ^#^ No confidence interval available.

Subgroup/Cohort	Medication	12 Months Prior, Observed	6 Months after, Estimated, 95% CI
Total cohort (N = 80,426)	SAPT, n (%)	20,594 (25.6)	37.9 (37.6–38.3)
	DAPT, n (%)	3330 (4.1)	14.8 (14.6–15.1)
	VKA, n (%)	7317 (9.1)	7.5 (7.3–7.7)
	DOAC, n (%)	3166 (3.9)	4.3 (4.1–4.4)
	DPI, n (%)	3116 (3.9)	7.4 (7.2–7.6)
	No therapy, n (%)	42,903 (53.3)	28.1 ^#^
CLTI (N = 40,004)	SAPT, n (%)	9828 (24.6)	34.3 (33.9–34.8)
	DAPT, n (%)	1456 (3.6)	12.3 (11.9–12.6)
	VKA, n (%)	5105 (12.8)	10.3 (10.0–10.7)
	DOAC, n (%)	2120 (5.3)	6.1 (5.9–6.4)
	DPI, n (%)	2013 (5.0)	9.3 (9–9.6)
	No therapy, n (%)	19,482 (48.7)	27.7 ^#^
IC (N = 40,422)	SAPT, n (%)	10,766 (26.6)	41.3 (40.8–41.8)
	DAPT, n (%)	1874 (4.6)	17.2 (16.9–17.6)
	VKA, n (%)	2212 (5.5)	5.0 (4.8–5.2)
	DOAC, n (%)	1046 (2.6)	2.7 (2.5–2.8)
	DPI, n (%)	1103 (2.7)	5.6 (5.4–5.8)
	No therapy, n (%)	23,421 (57.9)	28.2 ^#^

## Data Availability

Not applicable.

## Supplementary Materials

The following supporting information can be downloaded at: www.mdpi.com/article/10.3390/jcm11185455/s1, Table S1: Codings used for identifying risk factors, comorbidities, prior diagnoses and in-hospital procedures; Table S2: Baseline characteristics of the total study cohort and CLTI and IC patient, excluding patients suffering from atrial fibrillation within 1 year prior to index date, venous thromboembolism within 60 days prior to the index date, acute coronary syndrome within 1 year prior to index date and hip or knee replacement within 60 days prior to index date; SD: Standard Deviation; PAOD: Peripheral arterial occlusive disease; TIA: Transient ischaemic attack; CLTI: Chronic limb-threatening ischaemia, IC: Intermittent Claudication; Table S3: Observed and estimated treatment patterns of the total study cohort (a), CLTI (b) and IC (c) patients, excluding patients suffering from atrial fibrillation within 1 year prior to index date, venous thromboembolism within 60 days prior to the index date, acute coronary syndrome within 1 year prior to index date and hip or knee replacement within 60 days prior to index date; SAPT: Single antiplatelet therapy, DAPT: Dual antiplatelet therapy, VKA: Vitamin-K-antagonists, DOAC: Direct oral anticoagulation, DPI: Dual pathway inhibition, IC: Intermittent claudication, CLTI: Chronic limb-threatening ischaemia, CI: Confidence interval), # No confidence interval available; Table S4: Prior studies on the prescription of antithrombotics in patients with Peripheral arterial occlusive disease (PAOD)1-17; IC: Intermittent claudication, CLTI: Chronic limb-threatening ischaemia, CVD: Cardiovascular disease, MI: Myocardial infarction, APT: Antiplatelet therapy, OAC: Oral anticoagulation; we estimated upper bounds of antiplatelet use if medication use was not assessed mutually exclusive.
