# Supplementary material for: Antithrombotic Treatment Patterns of Patients with Symptomatic Peripheral Arterial Occlusive Disease in Germany: Evidence from Health Insurance Claims Data
Source: J Clin Med. 2022 Sep 16;11(18):5455. doi: 10.3390/jcm11185455 (PMC9501602; doi:10.3390/jcm11185455)
Supplement: Supplementary file 1 [file jcm-11-05455-s001.zip › jcm-1891801-supplementary.pdf]

Electronic Supplement

Table S1. Codings used for identifying risk factors, comorbidities, prior diagnoses and in-hospital procedures.

	**International Classification of Diseases 10th revision with German Modifications (ICD-10 GM) code**	**Anatomical Therapeutic Chemical (ATC) code**	**Operations and Procedures (OPS) code**
**Study population**			
Peripheral arterial occlusive disease (PAOD)	Main diagnosis: <2015 I70.21-24 ; ≥2015 I70.21-25 or main diagnosis E10.50-51, E10.7, E11.50-51, E11.7, I73.0 I73.1 I73.8-9 I74.0-5 I74.8-9 L03.01-2 L03.11 L98.4 R02 in combination with secondary diagnosis <2015 I70.21-24 ; ≥2015 I70.21-25		
**Obtained in-hospital treatment**			
Peripheral vascular intervention			8-836 8-840 to 8-849 8-83C 8-84A
Open surgical repair			5-380 to 5-384 5-38C to 5-38F 5-393 to 5-396 5-98A 5-38A.4 5-38A.C
Minor or major amputation			5-864 5-865
**Medical history**			
Congestive heart failure	I09.0 I11.0 I13.0 I13.2 I25.5 I42.0 I42.5-9 I43 I50 P29		
Cardiac arrhythmias	I44.1-3 I45.6 I45.9 I47 I48 I49 R00.0 R00.1 R00.8 T82.1 Z45.0 Z95.0		
Valvular disease	A52.0 I05.0-2 I05.8-9 I06.0-2 I06.8-9 I07.0-2 I07.8-9 I08.0-3 I08.8-9 I09.1 I09.8 I34-I39 Q23 Z95.2-4		
Diabetes, complicated	E10.2-8 E11.2-8 E12.2-8 E13.2-8 E14.2-8		
Renal failure	I12.0 I13.1 N18 N19 N15.0 Z49 Z94.0 Z99.2		
Liver disease	B18.0-2 B18.8-9 I85 I86.4 I98.2 K70 K71-74 K76 Z94.4		
Tumour/metastatic cancer	C00-C26 C30-C76 C97/C77-C80		
Dyslipidaemia	E78		
Ischemic heart disease	I20-I25		
Myocardial infarction	I20.0 I21-I24 Z03.4		
Stroke or TIA	I61 I63 I64 G45		
Venous Thromboembolism	I26, I80, I81, I82.2, I82.9		
Atrial Fibrillation	I48		
Hip or knee replacement			5-820 to 5-823
**Medications**			
Group 1, SAPT: Clopidogrel		B01AC04	

Group 1, SAPT: ASS		B01AC06<br>B01AC56<br>B01AC86	
Group 2, DAPT: Clopidogrel + ASS, dual		B01AC34	
Group 3, VKA: Phenprocoumon		B01AA04	
Group 3, VKA: Warfarin		B01AA03	
Group 4, DOAC: Dabigatran		B01AE07	
Group 4, DOAC: Rivaroxaban		B01AF01	
Group 4, DOAC: Apixaban		B01AF02	
Group 4, DOAC: Edoxaban		B01AF03	

Table S2. Baseline characteristics of the total study cohort and CLTI and IC patient, excluding patients suffering from atrial fibrillation within 1 year prior to index date, venous thromboembolism within 60 days prior to the index date, acute coronary syndrome within 1 year prior to index date and hip or knee replacement within 60 days prior to index date; SD: Standard Deviation; PAOD: Peripheral arterial occlusive disease; TIA: Transient ischaemic attack; CLTI: Chronic limb-threatening ischaemia, IC: Intermittent Claudication

	**Total (n=64,143)**	**CLTI (n=28,234)**	**IC (n=35,909)**
**Age**, years, mean (SD)	71.16 (11.20)	74.28 (11.79)	68.71 (10.05)
**Female** sex, n (%)	29520 (46.0)	13727 (48.6)	15793 (44.0)
**van Walraven Score** > 9, n (%)	19140 (29.8)	12090 (42.8)	7050 (19.6)
**Best medical treatment**, n (%)	12603 (19.6)	7849 (27.8)	4754 (13.2)
**Peripheral vascular intervention**, n (%)	33052 (51.5)	10819 (38.3)	22233 (61.9)
**Open surgical repair**, n (%)	15808 (24.6)	6963 (24.7)	8845 (24.6)
**Amputation only**, n (%)	2680 (4.2)	2603 (9.2)	77 (0.2)
**Congestive heart failure**, n (%)	13354 (20.8)	8411 (29.8)	4943 (13.8)
**Cardiac arrhythmias**, n (%)	8360 (13.0)	4843 (17.2)	3517 (9.8)
**Valvular disease**, n (%)	5160 (8.0)	3048 (10.8)	2112 (5.9)
**Hypertension**, n (%)	51205 (79.8)	23126 (81.9)	28079 (78.2)
**Diabetes (complicated)**, n (%)	16793 (26.2)	11313 (40.1)	5480 (15.3)
**Renal failure**, n (%)	16767 (26.1)	10135 (35.9)	6632 (18.5)
**Liver disease**, n (%)	2807 (4.4)	1603 (5.7)	1204 (3.4)
**Solid tumor or metastatic cancer**, n (%)	4732 (7.4)	2359 (8.4)	2373 (6.6)
**Dyslipidaemia**, n (%)	30524 (47.6)	12121 (42.9)	18403 (51.2)
History of **coronary artery disease**, n (%)	20542 (32.0)	9697 (34.3)	10845 (30.2)
Prior **myocardial infarction**, n (%)	7961 (12.4)	3818 (13.5)	4143 (11.5)
Prior **stroke** or **TIA**, n (%)	5155 (8.0)	3042 (10.8)	2113 (5.9)
History of **bleeding**, n (%)	13386 (20.9)	8251 (29.2)	5135 (14.3)

Table S3: Observed and estimated treatment patterns of the total study cohort (a), CLTI (b) and IC (c) patients, excluding patients suffering from atrial fibrillation within 1 year prior to index date, venous thromboembolism within 60 days prior to the index date, acute coronary syndrome within 1 year prior to index date and hip or knee replacement within 60 days prior to index date; SAPT: Single antiplatelet therapy, DAPT: Dual antiplatelet therapy, VKA: Vitamin-K-antagonists, DOAC: Direct oral anticoagulation, DPI: Dual pathway inhibition, IC: Intermittent claudication, CLTI: Chronic limb-threatening ischaemia, CI: Confidence interval), #No confidence interval available

(a)

**Total (n=64,143)**	**12 months prior, observed**	**6 months after, estimated,95% CI**
**SAPT**, n (%)	18026 (28.1)	42.3 (41.9-42.7)
**DAPT**, n (%)	2872 (4.5)	16.8 (16.6-17.1)
**VKA**, n (%)	2167 (3.4)	3.9 (3.7-4.0)
**DOAC**, n (%)	764 (1.2)	1.6 (1.5-1.7)
**DPI**, n (%)	843 (1.3)	3.9 (3.8-4.1)
**No therapy**, n (%)	39471 (61.5)	31.5#

(b)

**CLTI (n=28,234)**	**12 months prior, observed**	**6 months after, estimated,95% CI**
**SAPT**, n (%)	7894 (28)	39.6 (39-40.2)
**DAPT**, n (%)	1130 (4.0)	14.5 (14.1-15)
**VKA**, n (%)	1374 (4.9)	5.5 (5.2-5.8)
**DOAC**, n (%)	469 (1.7)	2.2 (2.1-2.4)
**DPI**, n (%)	468 (1.7)	5.2 (5-5.5)
**No therapy**, n (%)	16899 (59.9)	33.0#

(c)

**IC (n=35,909)**	**12 months prior, observed**	**6 months after, estimated,95% CI**
**SAPT**, n (%)	10132 (28.2)	44.4 (43.9-44.9)
**DAPT**, n (%)	1742 (4.9)	18.6 (18.2-19)
**VKA**, n (%)	793 (2.2)	2.7 (2.5-2.8)
**DOAC**, n (%)	295 (0.8)	1.1 (1.0-1.2)
**DPI**, n (%)	375 (1.0)	2.9 (2.7-3.1)
**No therapy**, n (%)	22572 (62.9)	30.4#

Table S4: Prior studies on the prescription of antithrombotics in patients with Peripheral arterial occlusive disease (PAOD) [1–17]; IC: Intermittent claudication , CLTI: Chronic limb-threatening ischaemia, CVD: Cardiovascular disease, MI: Myocardial infarction, APT: Antiplatelet therapy, OAC: Oral anticoagulation; we estimated upper bounds of antiplatelet use if medication use was not assessed mutually exclusive

Author	Year	Country	Patient subgroups	N	Female	Mean age	APT	OAC
Lee et al. [1]	2020	KR	PAOD	765	18%	67	98%	-
Hageman et al. [2]	2020	NL	PAOD, CVD	1,455	32%	60	55%	14%
Mustapha et al. [3]	2020	US	CLTI	404	33%	69	92%	11%
Galas et al. [4]	2019	DE	PAOD, nursing home	1,329	71%	84	≤ 41%	15%
Sigvant et al. [5]	2019	SE	MI, PAOD	52,408	49%	74	≤ 72%	-
Arruda-Olson et al. [7]	2018	US	PAOD	1,676	45%	72	60%	-
Cea Soriano et al. [9]	2017	UK	symptomatic PAOD	28,484	40%	-	≤ 23%	-
Sigvant et al. [10]	2016	SE	PAOD	18,742	49%	74	≤ 73%	-
Hussain et al. [11]	2016	CA	PAOD	2,686	35%	70	67%	15%
Coveney et al. [12]	2011	IE	Arterial disease	180	21%	69	≤ 87%	8%
Müller-Bühl et al. [13]	2011	DE	PAOD	479	44%	47	≤ 64%	12%
Pande et al. [14]	2011	US	PAOD	7,458	58%	68	39%	-
Stansby et al. [15]	2011	UK	IC	473	34%	68	70%	4%
Soga et al. [16]	2010	JP	IC	271	18%	68	92%	-
Paquet et al. [17]	2010	CA	PAOD	5,962	44%	73	72%	-
**Our study**	**2020**	**DE**	**PAOD**	**80,426**	**46%**	**73**	**60%**	**19%**

References

1. Lee, M.S.; Choi, B.G.; Rha, S.W. Impact of diabetes mellitus on 5-year clinical outcomes following successful endovascular revascularization for peripheral artery disease. *Vasc Med* **2020**, *25*, 33-40, doi:10.1177/1358863x19879751.
2. Hageman, S.H.J.; de Borst, G.J.; Dorresteijn, J.A.N.; Bots, M.L.; Westerink, J.; Asselbergs, F.W.; Visseren, F.L.J. Cardiovascular risk factors and the risk of major adverse limb events in patients with symptomatic cardiovascular disease. *Heart* **2020**, 10.1136/heartjnl-2019-316088, doi:10.1136/heartjnl-2019-316088.
3. Mustapha, J.; Gray, W.; Martinsen, B.J.; Bolduan, R.W.; Adams, G.L.; Ansel, G.; Jaff, M.R. One-Year Results of the LIBERTY 360 Study: Evaluation of Acute and Midterm Clinical Outcomes of Peripheral Endovascular Device Interventions. *J Endovasc Ther* **2020**, *26*, 143-154, doi:10.1177/1526602819827295.
4. Galas, N.; Becker, I.; Ficon, T.; Sakrauski, M.; Reichert, R.; Ahmad, W.; Mylonas, S.; Brunkwall, J.; Majd, P. Prescription rate of anti-atherosclerotic drugs in German nursing homes and its impact on outcome. *Vasa* **2019**, *48*, 158-166, doi:10.1024/0301-1526/a000754.
5. Sigvant, B.; Hasvold, P.; Thuresson, M.; Jernberg, T.; Janzon, M.; Nordanstig, J. Myocardial infarction and peripheral arterial disease: Treatment patterns and long-term outcome in men and women results from a Swedish nationwide study. *Eur J Prev Cardiol* **2019**, 10.1177/2047487319893046, 2047487319893046, doi:10.1177/2047487319893046.
6. Kawarada, O.; Nakai, M.; Nishimura, K.; Miwa, H.; Iwasaki, Y.; Kanno, D.; Nakama, T.; Yamamoto, Y.; Ogata, N.; Nakamura, M.; et al. Antithrombotic therapy after femoropopliteal artery stenting: 12-month results from Japan Postmarketing Surveillance. *Heart Asia* **2019**, *11*, e011114, doi:10.1136/heartasia-2018-011114.
7. Arruda-Olson, A.M.; Afzal, N.; Priya Mallipeddi, V.; Said, A.; Moussa Pacha, H.; Moon, S.; Chaudhry, A.P.; Scott, C.G.; Bailey, K.R.; Rooke, T.W.; et al. Leveraging the Electronic Health Record to Create an Automated Real-Time Prognostic Tool for Peripheral Arterial Disease. *J Am Heart Assoc* **2018**, *7*, e009680, doi:10.1161/jaha.118.009680.
8. Norgren, L.; Patel, M.R.; Hiatt, W.R.; Wojdyla, D.M.; Fowkes, F.G.R.; Baumgartner, I.; Mahaffey, K.W.; Berger, J.S.; Jones, W.S.; Katona, B.G.; et al. Outcomes of Patients with Critical Limb Ischaemia in the EUCLID Trial. *Eur J Vasc Endovasc Surg* **2018**, *55*, 109-117, doi:10.1016/j.ejvs.2017.11.006.
9. Cea Soriano, L.; Fowkes, F.G.R.; Johansson, S.; Allum, A.M.; Garcia Rodriguez, L.A. Cardiovascular outcomes for patients with symptomatic peripheral artery disease: A cohort study in The Health Improvement Network (THIN) in the UK. *Eur J Prev Cardiol* **2017**, *24*, 1927-1937, doi:10.1177/2047487317736824.
10. Sigvant, B.; Kragsterman, B.; Falkenberg, M.; Hasvold, P.; Johansson, S.; Thuresson, M.; Nordanstig, J. Contemporary cardiovascular risk and secondary preventive drug treatment patterns in peripheral artery disease patients undergoing revascularization. *J Vasc Surg* **2016**, *64*, 1009-1017.e1003, doi:10.1016/j.jvs.2016.03.429.
11. Hussain, M.A.; Al-Omran, M.; Mamdani, M.; Eisenberg, N.; Premji, A.; Saldanha, L.; Wang, X.; Verma, S.; Lindsay, T.F. Efficacy of a Guideline-Recommended Risk-Reduction Program to Improve Cardiovascular and Limb Outcomes in Patients With Peripheral Arterial Disease. *JAMA Surg* **2016**, *151*, 742-750, doi:10.1001/jamasurg.2016.0415.
12. Coveney, A.P.; O'Brien, G.C.; Fulton, G.J. ACE up the sleeve - are vascular patients medically optimized? *Vasc Health Risk Manag* **2011**, *7*, 15-21, doi:10.2147/vhrm.S15484.
13. Muller-Buhl, U.; Laux, G.; Szecsenyi, J. Secondary Pharmacotherapeutic Prevention among German Primary Care Patients with Peripheral Arterial Disease. *Int J Vasc Med* **2011**, *2011*, 316496, doi:10.1155/2011/316496.
14. Pande, R.L.; Perlstein, T.S.; Beckman, J.A.; Creager, M.A. Secondary prevention and mortality in peripheral artery disease: National Health and Nutrition Examination Study, 1999 to 2004. *Circulation* **2011**, *124*, 17-23, doi:10.1161/circulationaha.110.003954.

15. Stansby, G.; Mister, R.; Fowkes, G.; Roughton, M.; Nugara, F.; Brittenden, J.; Bradbury, A.; Ashley, S.; Shearman, C.; Hannon, R.; et al. High risk of peripheral arterial disease in the United Kingdom: 2-year results of a prospective registry. *Angiology* **2011**, *62*, 111-118, doi:10.1177/0003319710387917.
16. Soga, Y.; Yokoi, H.; Urakawa, T.; Tosaka, A.; Iwabuchi, M.; Nobuyoshi, M. Long-term clinical outcome after endovascular treatment in patients with intermittent claudication due to iliofemoral artery disease. *Circ J* **2010**, *74*, 1689-1695, doi:10.1253/circj.cj-10-0077.
17. Paquet, M.; Pilon, D.; Tetrault, J.P.; Carrier, N. Protective vascular treatment of patients with peripheral arterial disease: guideline adherence according to year, age and gender. *Can J Public Health* **2010**, *101*, 96-100.
